# Adenosine-Sensitive Upper Septal Fascicular Ventricular Tachycardia Associated With Takotsubo Syndrome

**DOI:** 10.1016/j.jaccas.2024.102811

**Published:** 2025-01-15

**Authors:** Parker O’Neill, Daniel Underberg, Reginald Ho, Darius Farzad, Gregary D. Marhefka

**Affiliations:** a Division of Cardiology, Thomas Jefferson University Hospital, Philadelphia, Pennsylvania, USA; b Department of Medicine, Thomas Jefferson University Hospital, Philadelphia, Pennsylvania, USA

**Keywords:** cardiomyopathy, cardioversion, ventricular tachycardia

## Abstract

We describe a rare case of upper septal fascicular ventricular tachycardia (VT) associated with takotsubo syndrome that failed to convert with synchronized shock but converted to normal sinus rhythm after intravenous adenosine administration. The excess catecholamine state of takotsubo syndrome likely provided substrate for cyclic adenosine monophosphate–mediated triggered activity, causing fascicular VT.

## Graphical Abstract

**Figure:**
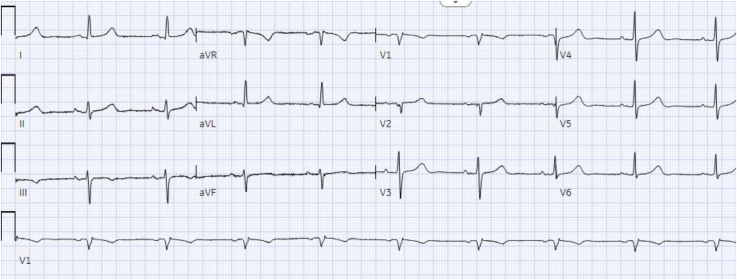

## History of Presentation

An 83-year-old woman with takotsubo syndrome was admitted to the cardiac intensive care unit for management of recurrent wide QRS complex tachycardia (WCT) with hemodynamic instability. The patient was admitted 2 days earlier with sudden onset chest pain after receiving an intraorbital anti–vascular endothelial growth factor injection for macular degeneration. Her electrocardiogram (ECG) on admission showed ST-segment elevation in leads V_2_ to V_4_ and an elevated high-sensitivity troponin level of 370 ng/L. A limited transthoracic echocardiogram (TTE) in the emergency department showed segmental wall motion abnormalities in the anteroseptal and inferolateral regions concerning for possible left anterior descending artery infarct. She was urgently taken to the catheterization laboratory. Cardiac catheterization showed only luminal irregularities and minimal coronary artery disease. Results of a formal TTE were consistent with takotsubo syndrome with apical ballooning and an ejection fraction of 30 ± 5%. While in the hospital she developed multiple episodes of WCT with associated hypotension that recurred despite the initiation of intravenous (IV) procainamide. The patient was alert and asymptomatic during these episodes of tachyarrhythmia.

**Take-Home Messages**

• Takotsubo syndrome can precipitate VT.
• This VT frequently resolves on resolution of the cardiomyopathy.

## Past Medical History

Notably, 1 week before this hospitalization, findings on TTE performed for atrial fibrillation monitoring were grossly normal, with an ejection fraction of 64%, normal biventricular size and function, a moderately dilated left atrium (left atrial volume index, 44 mL/m^2^), and no valvular disease. Other medical problems included paroxysmal atrial fibrillation, hyperlipidemia, hypertension, chronic kidney disease stage III, age-related macular degeneration, and iron deficiency anemia.

## Differential Diagnosis

The differential diagnosis of the WCT was between ventricular tachycardia (VT) and supraventricular tachycardia (SVT) with aberrancy.

## Investigations

**Figure 1:**
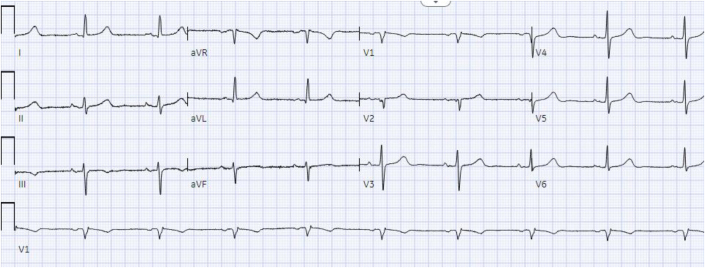
Baseline Electrocardiogram 1 Month Before Hospitalization

**Figure 2:**
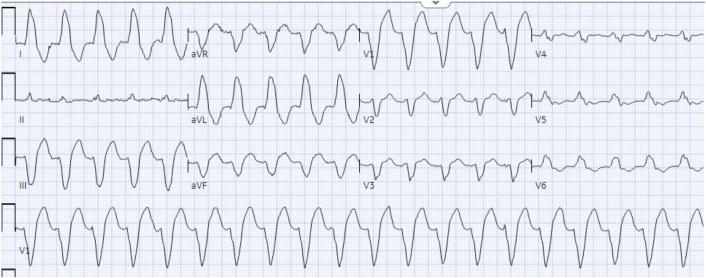
Electrocardiogram Showing Wide QRS Complex Tachycardia

**Figure 3:**
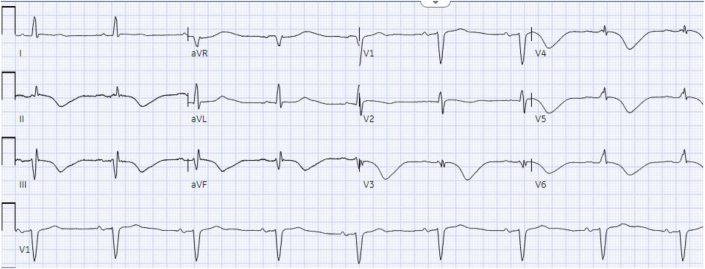
Electrocardiogram Between Wide Complex Tachycardia Episodes Showing Diffuse Large T-Wave Inversions With a Prolonged QT Interval

**Figure 4:**
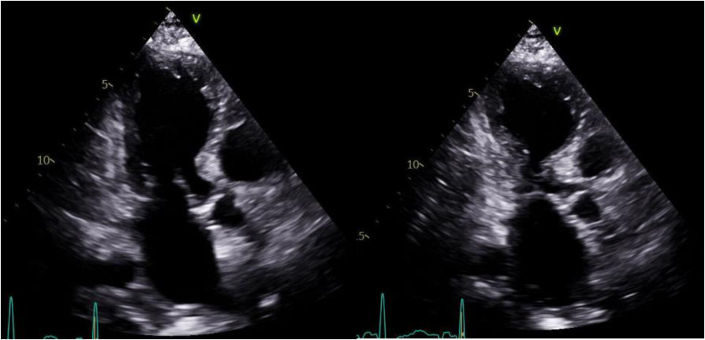
Transthoracic Echocardiogram (Left) Apical 3-chamber view showing diastole) compared with (right) systolic apical ballooning of the left ventricle.

The patient’s baseline ECG 1 month before admission, performed for atrial fibrillation monitoring, demonstrated sinus bradycardia at 55 beats/min and a QRS complex duration of 104 milliseconds (Figure 1). An ECG taken during an episode of WCT showed a regular wide complex monomorphic tachycardia at a rate of 120 beats/min, with a normal axis, a QRS complex duration of 196 milliseconds, a long intrinsicoid, and a left bundle branch block configuration pattern (Figure 2). An ECG obtained between WCT episodes showed sinus bradycardia at 51 beats/min with diffuse large T-wave inversions and with a QRS complex duration of 138 milliseconds and a QTc interval (Bazett) of 627 milliseconds (Figure 3). Laboratory tests revealed normal values of serum electrolytes, thyroid-stimulating hormone, renal function panel, and hemoglobin at baseline. The TTE on admission showed a newly reduced ejection fraction of 30% ± 5%, new periapical akinesis, and ballooning of the left ventricle suggestive of apical takotsubo syndrome (Figure 4).

## Management

**Figure 5:**
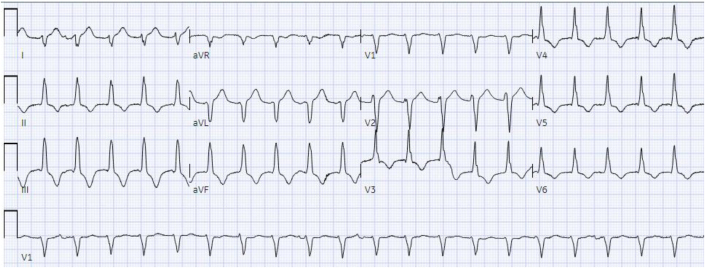
Electrocardiogram Showing Upper Septal Fascicular Ventricular Tachycardia

**Figure 6:**
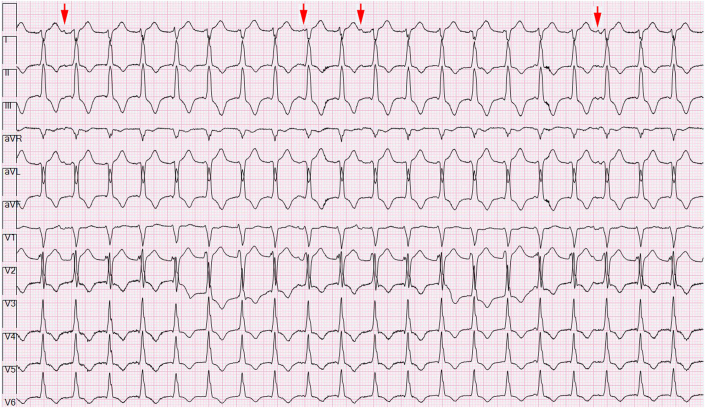
Same Electrocardiogram as in Figure 5 The 12-lead running rhythm strips with red arrows marking dissociated P waves in lead I.

**Figure 7:**
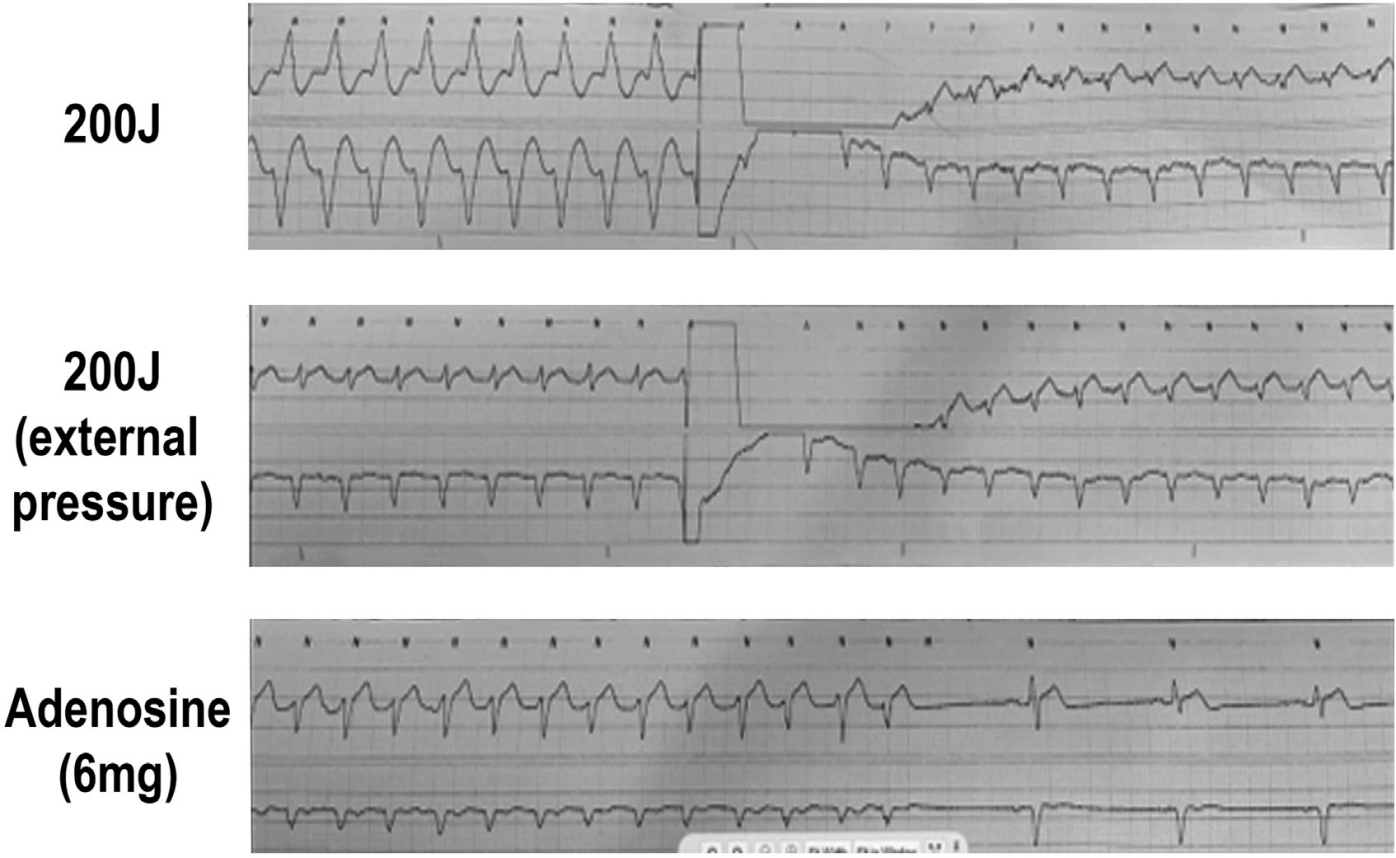
Telemetry Strip Showing Interventions (Cardioversion Twice and Adenosine Administration)

**Figure 8:**
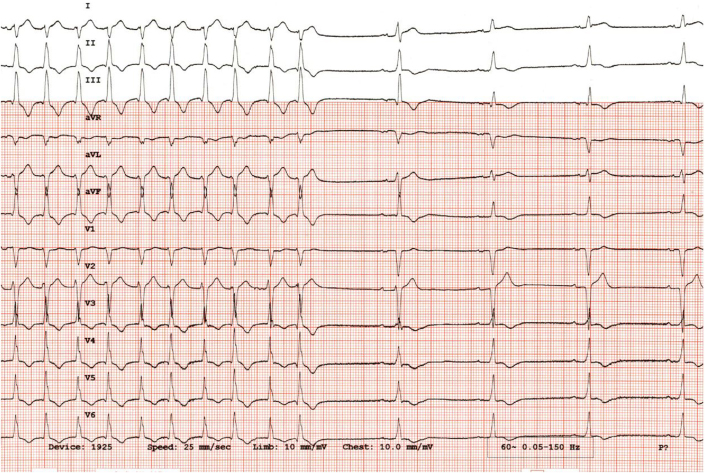
Telemetry Strip Showing Intravenous Adenosine (6 mg) Administration

**Figure 9:**
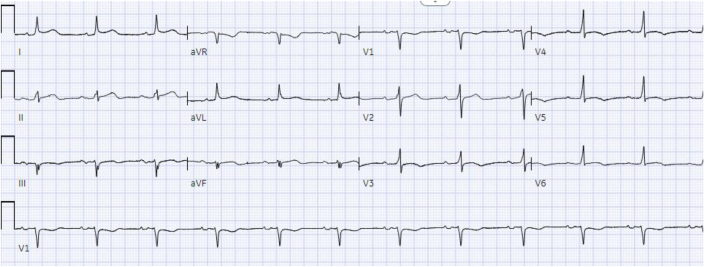
Electrocardiogram Showing Normal Sinus Rhythm After Administration of Intravenous Adenosine (6 mg)

The patient was initially monitored in the cardiac intensive care unit with IV procainamide and phenylephrine for blood pressure support. The WCT was thought to be monomorphic VT instead of SVT with aberrancy because of the long intrinsicoid, the prolonged QRS complex duration (196 milliseconds), and the precordial pattern break (V_2_R > V_3_R) atypical of left bundle aberration. However, the lack of atrioventricular (AV) dissociation with fusion or capture beats led to initial diagnostic uncertainty in the WCT diagnosis. When the persistent WCT did not resolve with medical management, the decision was made to perform electrical cardioversion. A 200-J biphasic synchronized shock was delivered that resulted in a change from the WCT (Figure 2) to a narrower complex tachycardia, which was interpreted as upper septal fascicular VT at a similar rate of 124 beats/min, with a rightward axis, and a QRS complex duration of 112 milliseconds (Figure 5). Atrioventricular dissociation with a slightly wider QRS complex duration and right-axis deviation compared with her baseline ECG favored a ventricular origin of the tachyarrhythmia (Figure 6). Although SVT with AV dissociation was a possibility, it is rare and would also have to be associated with a left posterior fascicular block at such a slow tachycardia rate (124 beats/min) to cause this clinical picture. A second 200-J shock delivered with external pressure applied did not change the rhythm. Subsequently, IV adenosine, 6 mg, was administered, which led to conversion to normal sinus rhythm with a rate of 68 beats/min, a normal axis, and a normal QRS complex duration of 93 milliseconds and nonspecific ST-segment and T-wave abnormalities (Figures 7, 8 and 9).

## Outcome and Follow-Up

After administration of adenosine, the patient remained in normal sinus rhythm, with no recurrence of upper septal fascicular VT, and she was discharged 1 week later. A repeat echocardiogram at the end of the hospitalization showed almost complete resolution of wall motion abnormalities with mild apical hypokinesis and an improved ejection fraction of 50% ± 5%. On discharge, she continued her home regimen of amiodarone, 200 mg daily (for atrial fibrillation), and remained in normal sinus rhythm at her 1-month postdischarge cardiology visit.

## Discussion

This report highlights a rare case of adenosine-sensitive upper septal fascicular VT associated with takotsubo syndrome. Upper septal fascicular VT is a rare form of idiopathic VT that is usually seen in younger patients without structural heart disease and carries a good prognosis. Idiopathic VT can originate from any location in the heart, with the most common locations being the right ventricular outflow tract, the left ventricular outflow tract, and the fascicles of the left bundle branch.^1^ The left bundle branch fascicular locations can be further classified into 3 types: 1) left posterior fascicular (accounting for 90% of idiopathic fascicular VT); 2) left anterior fascicular (accounting for 10% of idiopathic fascicular VT); and 3) upper septal fascicular (accounting for 1%-5% of idiopathic fascicular VT).

The mechanism of idiopathic fascicular VT is thought to be related to either re-entry circuits involving the His-Purkinje system or triggered activity within the fascicles.^2^ The finding that our patient’s arrhythmia resolved after administration of adenosine suggests an underlying cyclic adenosine monophosphate (cAMP)–mediated, calcium-dependent triggered activity in the upper septal fascicle.^3^ Previous electrophysiology studies have shown other forms of fascicular VT (namely, left posterior and left anterior fascicular) to be secondary to a re-entry pathway with a calcium-dependent zone of slow conduction in or near the fascicular system.^4^ Because idiopathic fascicular VT follows the native conduction pathway, it tends to have a narrower QRS complex (120-140 milliseconds) than typical wide complex VT, and as a result, it can be difficult to discern from SVT with aberrancy. Various algorithms have been created to assist with differentiation between VT and SVT with aberrancy, including the Brugada criteria and the VT score.^5^^,^^6^ In this case, Figure 6 displays dissociated, non-nonconducted P waves meeting the third Brugada criterion and indicating a sensitivity of 82% and a specificity of 98% of VT.^5^ Similarly, the VT score indicates a high probability of VT when applied to Figure 5 that demonstrates a lead II R-wave to peak time >50 milliseconds (52 milliseconds in this case), AV dissociation, and an R-wave >40 milliseconds in lead V_2_ (42 milliseconds in this case), thus indicating a 99.6% likelihood of VT.^6^ Although not present in this case, fusion or capture beats may also be present on ECGs and would suggest VT over SVT. Furthermore, fascicular VT tends to have the following specific configuration on the ECG: left posterior fascicular VT displays a right bundle branch block configuration with a left superior axis; left anterior fascicular VT displays a right bundle branch block configuration with a right inferior axis; and upper septal fascicular VT displays a normal QRS complex configuration (although rarely it can have a right bundle branch block configuration) with a normal axis.

Idiopathic fascicular VT (particularly left posterior and left anterior fascicular VT) typically responds well to verapamil, which inhibits calcium influx through the slow channels and terminates the re-entry circuit. Unfortunately, prophylactic oral verapamil is less effective at preventing recurrence than intravenous verapamil for short-term termination of idiopathic fascicular VT.^4^ Of note, verapamil should be used with caution in patients with hypotension or cardiomyopathy because it may worsen hemodynamic instability. Given the paucity of reported upper septal fascicular VT, the optimal treatment is unclear. If the underlying mechanism is the result of cAMP-mediated triggered activity, then adenosine should terminate the tachyarrhythmia, as in this case report. If upper septal fascicular VT is caused by a re-entry circuit with a calcium-dependent zone of slow conduction, then verapamil should precipitate conversion to sinus rhythm. For those patients who have recurrence of idiopathic fascicular VT despite medical management, catheter ablation has been reported to have an 85% to 95% success rate.^7^ Unlike other forms of VT, idiopathic fascicular VT is generally benign and does not require placement of an implantable cardioverter-defibrillator.

Takotsubo syndrome has been associated with an increased risk of atrial and ventricular arrhythmias that resolve on recovery of the cardiomyopathy.^8^ Takotsubo syndrome is thought to be secondary to sympathetic overdrive and excessive catecholamine release, which can either facilitate enhanced automaticity or induce delayed afterdepolarization and cause cAMP-mediated triggered activity. Alternatively, studies have shown patients with takotsubo syndrome have extensive myocardial edema during the acute phase that could facilitate re-entry, leading to VT similar to scarring or fibrosis in structural heart disease.^9^ A large prospective study by Stiermaier et al^10^ found that ventricular arrhythmias occurred in 10% of patients with takotsubo syndrome. Up to 50% of patients with takotsubo syndrome demonstrate a prolonged QT interval (as our patient did [Figure 3]), thereby causing increased dispersion of ventricular refractoriness and predisposing her to early afterdepolarization, triggered activity, and polymorphic VT, which is the most common form of VT in takotsubo syndrome.^10^^,^^11^ By contrast, our patient had monomorphic VT, and its resistance to cardioversion and termination by adenosine points to a mechanism of delayed afterdepolarization and cAMP-mediated triggered activity. The finding that our patient did not experience recurrence of fascicular VT after resolution of her takotsubo syndrome supports these hypotheses.

## Conclusions

Upper septal fascicular VT is a rare form of idiopathic VT that can be mistaken for SVT with aberrancy. The prognosis is excellent, and the condition generally can be managed medically with adenosine or verapamil, with refractory cases treated with catheter ablation. This case report highlights a rare case of adenosine-sensitive upper septal fascicular VT associated with takotsubo syndrome.

## Funding Support and Author Disclosures

The authors have reported that they have no relationships relevant to the contents of this paper to disclose.
